# Increased risk of deep vein thrombosis and surgical site infection in cemented total knee arthroplasty: A nationwide propensity score-matched study in Japan

**DOI:** 10.1007/s00402-025-05943-4

**Published:** 2025-06-02

**Authors:** Yu Mori, Kunio Tarasawa, Hidetatsu Tanaka, Masayuki Kamimura, Kento Harada, Naoko Mori, Kiyohide Fushimi, Toshimi Aizawa, Kenji Fujimori

**Affiliations:** 1 Department of Orthopaedic Surgery, Tohoku University Graduate School of Medicine, Sendai, Japan; 2 Department of Health Administration and Policy, Tohoku University Graduate School of Medicine, Sendai, Japan; 3 Department of Radiology, Akita University Graduate School of Medicine, Akita, Japan; 4 Department of Health Policy and Informatics, Institute of Science Tokyo, Tokyo, Japan

**Keywords:** Total knee arthroplasty, Bone cement, Complication, Propensity score matching, Nationwide database

## Abstract

**Introduction:**

Total knee arthroplasty (TKA) is a widely used treatment for advanced knee osteoarthritis. While cemented fixation is the standard technique, cementless fixation has demonstrated comparable long-term outcomes. However, the association between cement use and postoperative complications, including venous thromboembolism and surgical site infections, remains unclear, particularly in Japanese patients. This study aimed to investigate the incidence of postoperative complications in cemented and cementless TKA using a nationwide database.

**Methods:**

A nationwide cohort study was conducted using Japan’s DPC database from April 2016 to March 2023. Patients who underwent TKA were identified, and postoperative complications, including deep vein thrombosis, pulmonary embolism, pneumonia, cerebrovascular events, postoperative cognitive dysfunction, and surgical site infection, were analyzed. One-to-one propensity score (PS) matching was performed based on age, sex, body mass index, type of anesthesia, simultaneous bilateral surgery, Charlson comorbidity index, and comorbidities to ensure comparability. Statistical analyses included χ² tests, Student’s t-tests, and multivariate logistic regression analysis.

**Results:**

A total of 228,595 patients met the eligibility criteria, with 21,906 matched pairs in the cemented and cementless groups after PS matching. The incidence of deep vein thrombosis (OR: 1.231, 95% CI: 1.151–1.316, *p* < 0.0001) and surgical site infection (OR: 1.716, 95% CI: 1.420–2.073, *p* < 0.0001) was significantly higher in the cemented group. No significant differences were observed in pulmonary embolism or other complications.

**Conclusion:**

Cement application was associated with an increased risk of deep vein thrombosis and surgical site infection. These findings suggest that careful perioperative management may be warranted in patients undergoing cemented TKA.

## Introduction

Total knee arthroplasty (TKA) is a well-established treatment for advanced knee osteoarthritis. TKA has been reported to reduce pain and restore joint function [1, 2]. Multiple studies have demonstrated favorable outcomes, with 90% of patients achieving successful results at 20 years postoperatively [3–5]. TKA fixation methods include cementless and cemented fixation. Cemented fixation has been extensively studied and is considered the standard technique, with its efficacy supported by registry data [6, 7].

In cementless TKA, once osseointegration is established, the likelihood of implant loosening is extremely low, except in cases of dissolution or infection [8]. Furthermore, multiple studies have reported favorable 10-year survival rates for cementless TKA, demonstrating comparable outcomes to cemented TKA [9–12].

Among the potential surgical factors associated with an increased risk of venous thromboembolism in patients undergoing TKA, polymethylmethacrylate bone cement has been identified as a possible contributor. Although bone cement has been suggested as a risk factor for venous thromboembolism, its association remains a subject of debate [13, 14]. The use of cement has been shown to affect hemodynamics and activate a hypercoagulable state [15]. Consequently, several studies have indicated a potential association between cement use and an increased risk of venous thromboembolism; however, these concerns have not yet been fully investigated [16, 17]. Further investigation into the association between cement use and complication risk is necessary to establish the evidence through large-scale studies, such as nationwide database research.

The Japanese DPC database is a beneficial resource for investigating postoperative complications in large-scale cohort studies in orthopedic surgery [18–24]. However, the complication risks associated with cemented and cementless TKA in Japanese patients remain insufficiently studied. This study aims to utilize a nationwide database of Japanese patients who underwent TKA to compare the incidence of in-hospital complications between cemented and cementless procedures. We conducted a large-scale nationwide case-cohort study using Japan’s insurance database to evaluate the risk of complications in cemented TKA. This study compared the incidence of complications, including deep vein thrombosis, pulmonary embolism, pneumonia, cerebrovascular events, postoperative cognitive dysfunction, and surgical site infections, in cohorts matched for age, sex, body mass index (BMI), type of anesthesia, simultaneous bilateral surgery, Charlson comorbidity index, and comorbidities between cemented and cementless groups.

## Methods

### Study design

The study complied with the guidelines of the Declaration of Helsinki and received ethical approval from the Institutional Review Board of our institute. The data were retrospectively collected from Japan’s national DPC reimbursement system database [25]. Informed consent was comprehensively obtained from all patients upon admission, including their agreement to the proposed treatment methods and the use of data collected during their treatment for academic purposes. Furthermore, this study does not contain any information that could identify individual participants. The research was conducted from April 2016 to March 2023 as part of a nationwide survey of hospitals participating in the Japanese DPC system. During this period, approximately 1,100 hospitals consistently contributed medical records and received approval for inclusion in this study. Patients who underwent TKA at these hospitals were included in the analysis, providing a comprehensive overview of current practices and outcomes of TKA across Japan. This clinical study focused on patients who underwent TKA, specifically analyzing postoperative complications in the cemented TKA group compared with the cementless group. The postoperative complications assessed in this study included pulmonary embolism, deep vein thrombosis, cerebrovascular disease, postoperative cognitive dysfunction, pneumonia, surgical site infection, and periprosthetic fractures. Postoperative cognitive dysfunction was identified using the ICD-10 codes F010, F011, F012, F019, F03, F107, G238, G300, G301, G308, G309, G310, and G318, which encompass cognitive dysfunction and delirium occurring after surgery, as previously described [23]. The primary diagnoses for TKA were classified using ICD-10 codes, focusing on knee joint deformities caused by osteoarthritis and rheumatoid arthritis. Osteoarthritis was categorized under codes M170–M175 and M179, while rheumatoid arthritis was classified under codes M0586, M0606, M0686, and M0696. Additionally, the use of antithrombotic agents was systematically analyzed. The cohort of patients undergoing TKA, including both cemented and cementless procedures, was identified based on three primary criteria: (1) primary diagnosis, (2) main reason for admission, and (3) condition requiring the highest medical resource utilization. Cases of bilateral simultaneous surgery were included in the study, while patients who underwent revision TKA were excluded.

### Propensity score matching

This study compared postoperative complications between cemented TKA patients and those who underwent cementless TKA. A one-to-one propensity score (PS) matching approach was applied to ensure a balanced comparison between the two groups. The analysis accounted for confounding factors, including age, gender, BMI, type of anesthesia, simultaneous bilateral surgeries, hypertension, diabetes, cerebrovascular disease, ischemic heart disease, chronic renal dysfunction, chronic pulmonary disease, cognitive impairment, hyperlipidemia, rheumatoid arthritis, and other comorbidities, by utilizing the Charlson comorbidity index. The model’s discriminatory ability was evaluated using the C statistic. PS estimates were applied for nearest-neighbor matching without replacement, with a caliper width set to 0.2 times the standard deviation of the PS estimate [18]. This approach yielded a balanced cohort of patients undergoing cemented and cementless TKA based on PS matching, ensuring comparability between groups and providing matched pairs for analysis.

### Statistical analyses

Data are presented as mean ± standard deviation. Differences in clinical parameters between the cemented and cementless groups were assessed using the χ² test and Student’s t-test both before and after PS matching. The risk of postoperative complications between the groups was also evaluated using the χ² test and Student’s t-test before and after PS matching. For significant severe postoperative complications and in-hospital mortality, multivariate logistic regression analysis was performed to identify independent risk factors, including factors beyond cement application. This approach identified independent risk factors associated with these complications. Due to the large sample size in this study, a stricter threshold for statistical significance was applied. All statistical tests were two-sided, with statistical significance set at a p-value of less than 0.001. Statistical analyses were conducted using JMP version 17 (SAS, Cary, NC).

## Results

**Fig. 1:**
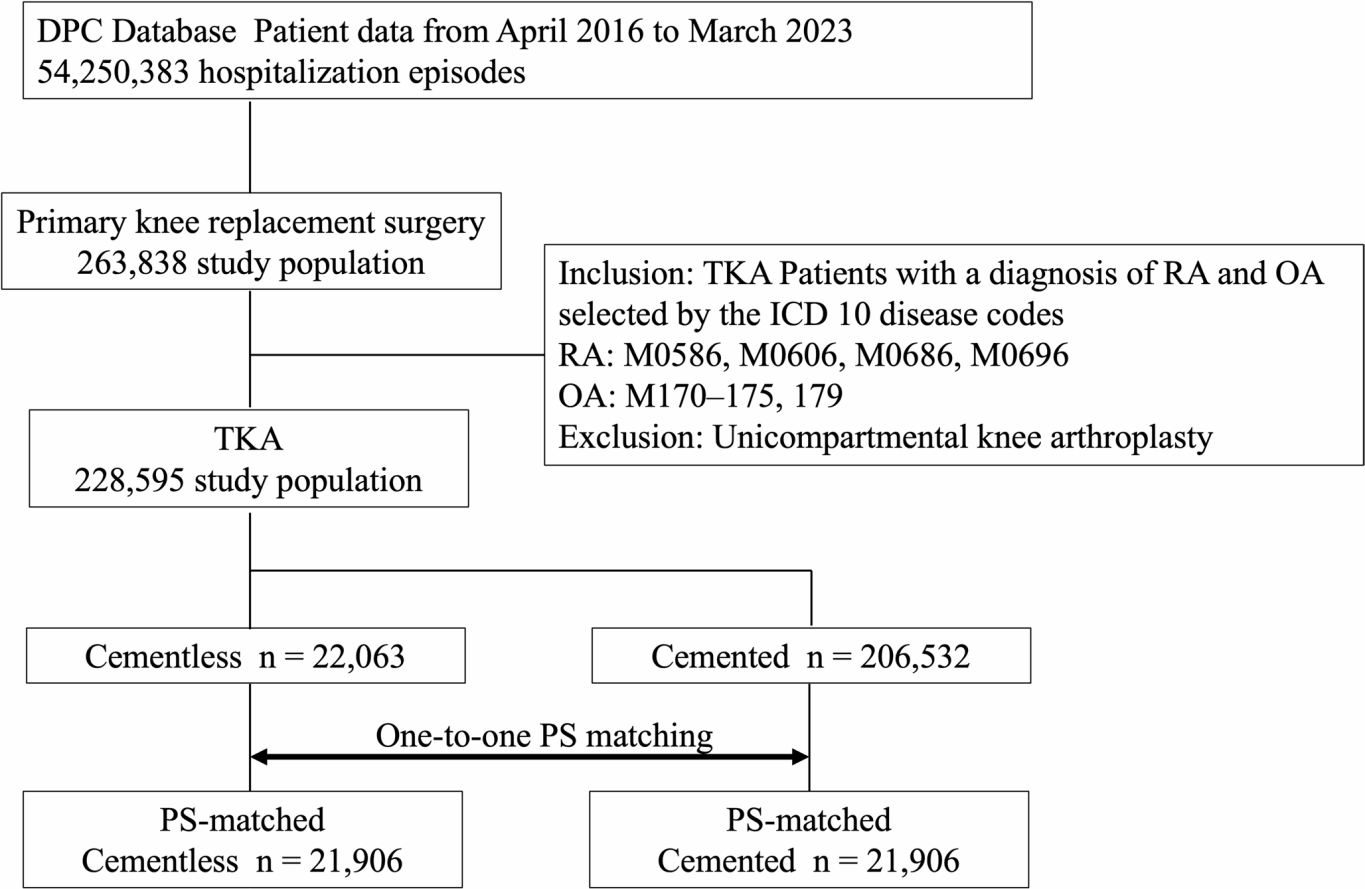
The flow diagram depicts the patient selection process for the cemented and cementless groups undergoing knee replacement surgery for osteoarthritis (OA) or rheumatoid arthritis (RA), along with the propensity score (PS) matching procedure. It outlines the methodology for extracting target patients from the Diagnosis Procedure Combination (DPC) database and the subsequent PS matching process for the cemented and cementless TKA cohorts

Figure 1 presents a schematic representation of the patient selection process. From a dataset covering April 2016 to March 2023, a total of 228,595 patients met the eligibility and exclusion criteria. Among them, 206,532 were classified into the cemented group, while 22,063 were in the cementless group. Following PS matching, which accounted for age, sex, BMI, type of anesthesia, simultaneous bilateral surgery, Charlson comorbidity index, and comorbidities, the cemented and cementless groups comprised 21,906 patients.

Table 1 summarizes the characteristics of patients in the cemented and cementless groups who underwent TKA. Before PS matching, the two groups exhibited significant differences in sex, BMI, Charlson comorbidity index, the prevalence of hypertension, hyperlipidemia, rheumatoid arthritis, and the frequency of general anesthesia and simultaneous bilateral surgery. The cemented group had a higher average age, a higher proportion of female patients, a lower BMI, a higher Charlson comorbidity index, and a lower prevalence of hypertension, and hyperlipidemia. In contrast, rheumatoid arthritis and general anesthesia were more prevalent in the cemented group. Additionally, the frequency of simultaneous bilateral surgery was higher in the cemented group. After a one-to-one PS matching, the standardized mean difference (SMD) for all variables was less than 0.1, demonstrating that these factors were appropriately balanced. The length of hospital stay was significantly longer in the cemented group compared to the cementless group. In contrast, the amount of blood transfused on the day of surgery and the following day showed no significant difference between the two groups. These findings were consistent before and after PS matching. The C-statistic was calculated to be 0.774.

**Table 1:** Characteristics of patients before and after propensity score matching

Covariates	Before PS matching			After PS matching			*p*-value
	Cementless	Cemented	*p*-value	Cementless	Cemented	SMD	
n	22,063	206,532		21,906	21,906		
Age	74.6 ± 7.7	74.8 ± 7.8	0.0022	74.6 ± 7.7	74.5 ± 7.9	0.009	0.29
Sex							
Male	5382 (24.4%)	42,457 (20.6%)	< 0.0001*	5348 (24.4%)	5508 (25.1%)	0.017	0.077
Female	16,681 (75.6%)	164,075 (79.4%)		16,558 (75.6%)	16,398 (74.9%)		
Body mass index	26.6 ± 4.3	26.1 ± 4.9	< 0.0001*	26.6 ± 4.3	26.5 ± 4.3	0.033	0.0004*
Hypertension	9872 (44.7%)	71,687 (34.7%)	< 0.0001*	9815 (44.8%)	9809 (44.8%)	0.0006	0.95
Diabetes	4610 (20.9%)	44,763 (21.7%)	0.008	4579 (20.9%)	4392 (20.1%)	0.021	0.027
Cerebrovascular disease	762 (3.4%)	6657 (3.2%)	0.066	762 (3.5%)	744 (3.4%)	0.005	0.64
Ischemic heart disease	1411 (6.4%)	12,204 (5.9%)	0.004	1404 (6.4%)	1346 (6.2%)	0.011	0.25
Chronic renal dysfunction	589 (2.7%)	5849 (2.8%)	0.17	587 (2.7%)	571 (2.6%)	0.005	0.63
Chronic lung disease	119 (0.5%)	1231 (0.6%)	0.29	119 (0.5%)	109 (0.5%)	0.006	0.51
Cognitive impairment	331 (1.5%)	3263 (1.6%)	0.37	327 (1.5%)	325 (1.5%)	0.0007	0.94
Hyperlipidemia	5211 (23.6%)	40,039 (19.4%)	< 0.0001*	5163 (23.6%)	5129 (23.4%)	0.004	0.71
Rheumatoid arthritis	707 (3.2%)	8398 (4.1%)	< 0.0001*	699 (3.2%)	685 (3.1%)	0.004	0.71
Charlson comorbidity index	0.68 ± 0.94	0.71 ± 0.95	< 0.0001*	0.68 ± 0.94	0.65 ± 0.92	0.027	0.004
General anesthesia	20,915 (94.8%)	187,638 (90.9%)	< 0.0001*	20,779 (94.9%)	20,714 (94.6%)	0.013	0.17
Simultaneous bilateral	1145 (5.2%)	14,230 (6.9%)	< 0.0001*	1142 (5.2%)	1122 (5.1%)	0.004	0.67
Non-covariates	Cementless	Cemented	*p*-value	Cementless	Cemented	F value or χ2 statics	*p*-value
Length of hospitalization (days)	27.1 ± 14.7	28.8 ± 16.0	< 0.0001*	27.1 ± 14.7	29.1 ± 14.9	192.6	< 0.0001*
Blood transfusion Day 0 (unit)	0.06 ± 0.40	0.06 ± 0.42	0.088	0.06 ± 0.40	0.06 ± 0.40	0.01	0.91
Blood transfusion Day 1 (unit)	0.03 ± 0.27	0.04 ± 0.30	< 0.0001*	0.03 ± 0.27	0.03 ± 0.27	1.7	0.19

One-to-one PS matching was performed

Data is shown as mean ± standard deviation; *p-values of < 0.001 are considered significant by the Student’s t-test and χ2 test difference

PS means propensity score; SMD means standard mean difference

Table 2 summarizes the use of anticoagulants and antiplatelet agents, which were administered not only for thromboprophylaxis but also for the management of comorbid conditions such as ischemic heart disease and cerebrovascular disease. Edoxaban was the most frequently used medication in both groups. Before PS matching, significant differences were observed between the two groups in the use of edoxaban, aspirin, warfarin, and apixaban. Edoxaban, aspirin, and warfarin were more commonly used in the cemented group, whereas apixaban was more frequently prescribed in the cementless group. After PS matching, edoxaban, aspirin, and warfarin were more frequently used in the cemented group, while apixaban was more commonly administered in the cementless group. Overall, anticoagulants and antiplatelet agents were appropriately utilized in both groups, primarily for the prevention of deep vein thrombosis and pulmonary embolism, although some patients may have received them for other indications such as cardiovascular or cerebrovascular diseases.

**Table 2:** Comparison of antithrombotic therapies before and after propensity score matching

	Before PS matching				After PS matching		
	Cementless	Cemented	*p*-value	Cementless	Cemented	χ2 statics	*p*-value
Edoxaban	12,228 (55.0%)	129,208 (62.6%)	< 0.0001*	12,140 (55.0%)	13,806 (63.0%)	262.3	< 0.0001*
Fondaparinux	421 (1.9%)	4105 (2.0%)	0.42	415 (1.9%)	465 (2.1%)	2.9	0.089
Enoxaparin	2121 (9.6%)	20,477 (9.9%)	0.15	2118 (9.7%)	2143 (9.8%)	0.2	0.69
Aspirin	1650 (7.5%)	17,309 (8.4%)	< 0.0001*	1641 (7.5%)	1853 (8.5%)	14.0	0.0002*
Warfarin	402 (1.8%)	4719 (2.3%)	< 0.0001*	400 (1.8%)	514 (2.3%)	14.5	< 0.0001*
Clopidogrel	629 (2.9%)	5684 (2.8%)	0.39	627 (2.9%)	623 (2.8%)	0.01	0.91
Apixaban	792 (3.6%)	5121 (2.5%)	< 0.0001*	788 (3.6%)	560 (2.6%)	39.8	< 0.0001*

One-to-one PS matching was performed

*p-values of < 0.001 are considered significant by the χ2 test; PS means propensity score

Table 3 presents the analysis results comparing the postoperative complication rates between the cemented and cementless groups. Before PS matching, the incidence of deep vein thrombosis and surgical site infection was significantly higher in the cemented group, while there was no significant difference in pulmonary embolism, cerebrovascular disorder, cognitive dysfunction, pneumonia, periprosthetic fracture, or in-hospital mortality between groups. After PS matching, a similar trend in complication rates persisted. Notably, the cemented group showed a pronounced tendency toward higher incidences of deep vein thrombosis and surgical site infection.

**Table 3:** Comparison of complications before and after propensity score matching

	Before PS matching			Cementless	After PS matching		
	Cementless	Cemented	*p*-value		Cemented	χ2 statics	*p*-value
Deep vein thrombosis	1732 (7.9%)	19,530 (9.5%)	< 0.0001*	1722 (7.9%)	2079 (9.5%)	36.7	< 0.0001*
Pulmonary embolism	79 (0.4%)	607 (0.3%)	0.097	79 (0.4%)	69 (0.3%)	0.7	0.41
Cerebrovascular disorder	68 (0.3%)	744 (0.4%)	0.22	68 (0.3%)	66 (0.3%)	0.03	0.86
Cognitive dysfunction	138 (0.6%)	1575 (0.8%)	0.025	138 (0.6%)	175 (0.8%)	4.4	0.036
Pneumonia	38 (0.2%)	406 (0.2%)	0.44	38 (0.2%)	34 (0.2%)	0.2	0.64
Surgical site infection	174 (0.8%)	2572 (1.3%)	< 0.0001*	172 (0.8%)	295 (1.4%)	32.7	< 0.0001*
Periprosthetic fracture	7 (0.03%)	132 (0.06%)	0.065	7 (0.03%)	15 (0.03%)	2.9	0.088
In-hospital mortality	7 (0.03%)	97 (0.05%)	0.31	7 (0.03%)	6 (0.03%)	0.08	0.78

One-to-one PS matching was performed

*p-values of < 0.001 are considered significant by the χ2 test; PS means propensity score

Table 4 presents the results of a multivariate logistic regression analysis examining factors associated with postoperative deep vein thrombosis following TKA. Female sex, cement application, and hypertension emerged as significant independent risk factors among the various factors examined. Female sex, cement application, and hypertension were identified as important risk factors for deep vein thrombosis, with an odds ratio of 1.216 (95% CI: 1.121–1.319, *p* < 0.0001), 1.231 (95% CI: 1.151–1.316, *p* < 0.0001), and 1.292 (95% CI: 1.204–1.386, *p* < 0.0001), respectively.

**Table 4:** Multivariate logistic analysis for risk factors for deep vein thrombosis after total knee arthroplasty during hospitalization

Variable	Odds Ratio (95% CI)	χ2 statics	*p*-value
Age	1.003 (0.998–1.007)	1.8	0.17
Sex (Female)	1.216 (1.121–1.319)	22.8	< 0.0001*
Bilateral surgery	1.068 (0.923–1.237)	0.8	0.37
Cement application	1.231 (1.151–1.316)	37.2	< 0.0001*
Hypertension	1.292 (1.204–1.386)	50.7	< 0.0001*
Diabetes	0.941 (0.865–1.023)	2.0	0.15
Cerebrovascular disease	0.899 (0.745–1.085)	1.3	0.27
Chronic renal dysfunction	0.911 (0.734–1.129)	0.7	0.39
Ischemic heart disease	0.903 (0.784–1.041)	2.0	0.16
Cognitive impairment	0.917 (0.692–1.215)	0.4	0.55
Chronic lung disease	1.081 (0.681–1.715)	0.1	0.74
Hyperlipidemia	1.065 (0.983–1.222)	2.4	0.12
Rheumatoid arthritis	1.009 (0.833–1.222)	0.01	0.92

*P-values of < 0.001 are considered significant by the χ2 test; CI means confidence interval

Table 5 presents the results of a multivariate logistic regression analysis examining factors associated with surgical site infection in patients undergoing TKA. Among the variables analyzed, cement application was identified as a significant independent risk factor, with an odds ratio of 1.716 (95% CI: 1.420–2.073, *p* < 0.0001) in this PS-matched cohort.

**Table 5:** Multivariate logistic analysis for risk factors for surgical site infection after total knee arthroplasty during hospitalization

Variable	Odds Ratio (95% CI)	χ2 statics	*p*-value
Age	0.989 (0.978–1.001)	3.1	0.077
Sex (Male)	1.356 (1.110–1.657)	8.6	0.0028
Bilateral surgery	1.046 (0.696–1.569)	0.05	0.83
Cement application	1.716 (1.420–2.073)	32.4	< 0.0001*
Hypertension	1.356 (1.117–1.646)	9.5	0.0021
Diabetes	1.075 (0.855–1.353)	0.4	0.53
Cerebrovascular disease	0.783 (0.457–1.341)	0.9	0.37
Chronic renal dysfunction	1.303 (0.798–2.126)	1.0	0.29
Ischemic heart disease	1.196 (0.849–1.685)	1.0	0.31
Cognitive impairment	2.142 (1.247–3.679)	6.2	0.0057
Chronic lung disease	2.614 (1.217–5.614)	4.6	0.14
Hyperlipidemia	1.205 (0.973–1.493)	2.9	0.087
Rheumatoid arthritis	1.236 (0.767–1.993)	0.7	0.38

*p-values of < 0.001 are considered significant by the χ2 test; CI means confidence interval

## Discussion

The results of this study revealed that, in a large-scale analysis adjusted for confounding factors, cemented TKA was associated with significantly higher odds ratios for postoperative complications compared with cementless TKA. Specifically, the odds ratio for postoperative deep vein thrombosis was 1.231 (95% CI: 1.151–1.316, *p* < 0.0001), while the odds ratio for surgical site infection was 1.716 (95% CI: 1.420–2.073, *p* < 0.0001). These findings indicate that the cement application is the independent risk factor in these complications. Conversely, cement application was not identified as a significant risk factor for pulmonary embolism or other complications. Additionally, this study examined the use of antithrombotic therapy, which was comparable between the cemented and cementless groups. While these agents were primarily administered for thromboprophylaxis, other indications such as cardiovascular or cerebrovascular conditions may also have contributed to their use. These findings underscore the importance of appropriate perioperative management in this population and provide valuable insights into the postoperative complications of cemented TKA, which have not been fully explored in previous reports.

The hypothesis that cement fixation of TKA increases the risk of pulmonary embolism and deep vein thrombosis has been proposed and remains a topic of debate [15, 26, 27]. It has been reported that cement activates the coagulation cascade intraoperatively, thereby increasing the risk of venous thromboembolism [27]. Our study identified cement fixation as a significant risk factor for deep vein thrombosis compared to the cementless group. These findings are consistent with previous studies that have reported cement as an important risk factor for venous thromboembolism [16, 17]. On the other hand, several studies have reported that the use of cement application in TKA is not associated with an increased risk of embolism or thrombosis [28, 29]. Although the scale is small, a meta-analysis comparing the incidence of deep vein thrombosis in cemented and cementless TKA found no significant difference between the two groups [29]. A large-scale study at a single institution comparing cemented and cementless TKA in 2382 cases, adjusted for confounding factors, also reported that cement application and tourniquet use were not significantly associated with deep vein thrombosis or pulmonary embolism. However, the study did not include information on the number of days spent in the hospital, and the authors also reported on the limitations of the study, including the possibility that subclinical deep vein thrombosis or pulmonary embolism could not be identified after discharge [28]. In this study, the average length of hospital stay was 29.1 ± 14.9 days in the cemented group and 27.1 ± 14.7 days in the cementless group, and the length of hospital stay in the cementless group was significantly shorter. The observation period was sufficient to monitor postoperative venous thrombosis during hospitalization; however, it may not fully capture complications occurring after discharge. The use of tourniquets is also sometimes discussed as a factor that raises concerns about deep vein thrombosis and pulmonary embolism [28, 29], so the use of cement and venous thrombosis will need to be examined in the future.

This study showed that cement application is an independent risk factor for surgical site infection. The first possible cause of this is the difference in operating time between cemented TKA and cementless TKA. With regard to the relationship between TKA surgery time and surgical site infection, previous reports have shown that an increase in the duration of surgery is associated with an increased risk of surgical site infection [30–32]. A meta-analysis of 427,361 cases reported that the odds ratio for surgical site infection was 1.50 when the duration of surgery exceeded 90 min [32]. On the other hand, previous research has shown that there is a significant difference in average operating time between cemented TKA (93.7 min) and uncemented TKA (82.1 min) [33]. Another meta-analysis also reported that the average operating time for cemented TKA is 81 min, while that for uncemented TKA is significantly shorter at 74 min [34]. Although we were unable to collect data on operating time in this study, previous research has shown that cemented TKA takes longer than cementless TKA, so it is thought that the use of cement is one of the factors that significantly contribute to the occurrence of surgical site infections.

There is also controversy over whether antibiotic-impregnated cement contributes to a reduction in the risk of infection [35]. In this study, we were unable to collect information on the type of cement used, and we were also unable to examine the use of antibiotics in combination. Whether the use of antibiotic-impregnated cement contributes to a reduction in surgical site infections should be a topic for future research.

A key strength of this study is its utilization of a nationwide database combined with PS matching, which effectively adjusts for confounding factors such as age, sex, body mass index, type of anesthesia, simultaneous bilateral surgery, Charlson comorbidity index, and comorbidities. Furthermore, the large sample size strengthens the statistical reliability of the findings. However, this study has several limitations, as detailed below. First, the study population was restricted to patients who underwent TKA in acute care hospitals participating in the DPC data system. This excludes patients admitted to non-DPC-reported beds, which comprise approximately 30% of all general hospital beds, as well as those who were never treated in an acute care hospital [36]. Secondly, this study is constrained by the inability to verify the accuracy of DPC disease classifications or assess the severity of symptoms associated with comorbidities in the actual patients. Thirdly, the study is further limited by its inability to confirm the severity of knee joint deformities or the details of the surgical approach and equipment utilized. The fourth limitation is that this study did not examine the duration of surgery, the use of tourniquets, or the use of antibiotic-mixed cement. Lastly, long-term outcomes were not assessed, including infection, periprosthetic fracture, reoperation, and mortality after discharge. Further large-scale studies incorporating more detailed patient data are warranted to overcome these limitations.

## Conclusion

This study found that cement application in TKA was associated with an increased risk of deep vein thrombosis and surgical site infection, with both identified as independent risk factors. In contrast, cement use did not appear to be significantly associated with pulmonary embolism or cerebrovascular events. Notably, the risk of deep vein thrombosis remained elevated despite the administration of anticoagulants and antiplatelet agents, which were used primarily for thromboprophylaxis but may have served other purposes as well. These findings indicate that careful postoperative monitoring may be advisable in patients undergoing cemented TKA, particularly with regard to thromboembolic complications and surgical site infections.

## Data Availability

The data supporting this study’s findings are available upon request from the corresponding author.

## Abbreviations

CI: Confidence interval
DPC: Diagnosis procedure combination
PS: Propensity score
SMD: Standard mean difference
TKA: Total knee arthroplasty
